# Data-driven approach for the prediction and interpretation of core-electron loss spectroscopy

**DOI:** 10.1038/s41598-018-30994-6

**Published:** 2018-09-06

**Authors:** Shin Kiyohara, Tomohiro Miyata, Koji Tsuda, Teruyasu Mizoguchi

**Affiliations:** 1 Institute of Industrial Science, The University of Tokyo, 153-8505 Tokyo, Japan; 2 Department of Computational Biology and Graduate School of Frontier Sciences, The University of Tokyo, Kashiwa, Chiba, 277-8561 Japan; 3 Center for Materials Research by Information Integration, National Institute for Materials Science, 1-2-1 Sengen, Tsukuba, 305-0047 Japan; 4 RIKEN Center for Advanced Intelligence Project, 1-4-1 Nihombashi Chuo-ku, 103-0027 Tokyo, Japan

## Abstract

Spectroscopy is indispensable for determining atomic configurations, chemical bondings, and vibrational behaviours, which are crucial information for materials development. Despite their importance, the interpretation of spectra using “human-driven” methods, such as the manual comparison of experimental spectra with reference/simulated spectra, is difficult due to the explosive increase in the number of experimental spectra to be observed. To overcome the limitations of the “human-driven” approach, we develop a new “data-driven” approach based on machine learning techniques by combining the layer clustering and decision tree methods. The proposed method is applied to the 46 oxygen-K edges of the ELNES/XANES spectra of oxide compounds. With this method, the spectra can be interpreted in accordance with the material information. Furthermore, we demonstrate that our method can predict spectral features from the material information. Our approach has the potential to provide information about a material that cannot be determined manually as well as predict a plausible spectrum from the geometric information alone.

## Introduction

Unveiling the atomistic configurations, chemical bondsing, and vibrational behaviours of molecules and atoms, which are commonly correlated to the functions of a material, is the most important task of materials research. Spectroscopic techniques, such as diffraction, reflection, emission, and absorption, have been used to identify such information, because the spectral features are correlated to the material information^1–4^. However, the spectrum does not directly provide the actual “values” of the material information. Thus, “interpretation” of the spectrum is necessary to determine the correlation between the spectral features and material information.

However, the interpretation of a spectrum is not always straightforward. For instance, X-ray diffraction and infrared absorption spectroscopies have been used for a long time in materials science as standard spectroscopic techniques for identifying crystal structures and the dynamical behaviours of molecules, respectively, and the observed spectra are typically interpreted by comparison to those in a database prepared from a long history of reference-compound observations. However, even in these well-known spectroscopic techniques, “unknown” features not present in the database are encountered in the spectra of “unknown” or “new” materials. Such cases are common in all spectroscopic techniques, and therefore, spectral simulations with suitable models are required.

Among existing spectroscopic techniques, core-loss spectroscopy using electrons or X-rays, namely, electron energy-loss near-edge structure (ELNES) and X-ray absorption near-edge structure (XANES), offer atomic-scale spatial resolution^5^, nanosecond-level time resolution^6^, and high sensitivity^7^ and can be considered to be the “ultimate analysis” in materials science^8^. As ELNES/XANES commonly originate from an electron transition from a core orbital to the conduction band (unoccupied orbitals), their spectral features reflect the atomic coordination, bond length, valence state, and local electronic structures; theoretical calculations have been used to interpret these spectral features^9–12^.

In ELNES/XANES experiments, time-resolved or/and spatially resolved observations are often performed. For instance, spatially resolved ELNES, namely, spectral imaging, with several hundreds of pixels has been reported^13–16^. In XANES, pico- or nanosecond time-resolved observations are possible, and time-resolved experiments for tracing chemical reactions have been performed^17–19^. Through such spatially/time-resolved ELNES/XANES observations, several thousands to tens of thousands of spectra can be observed in an experiment.

For a dataset containing numerous spectra, individual interpretation through theoretical calculations is unrealistic because each ELNES/XANES calculation requires exclusive knowledge of both the experiment and the theoretical calculation and involves many computations. Thus, the number of spectra that can be generated using these modern instruments cannot be handed by a “human-driven” interpretation approach.

Recently, approaches for obtaining new insights by handling big-data, called “data-driven” approaches, have attracted significant attention in materials science. By analysing big-data, these “data-driven” approaches have realized the discovery of desired structures with minimum computation^20–22^, new materials with higher performances^23–26^, and information that could not be previously determined from experiments^27,28^ and simulations^29,30^. Hence, this data-driven method has the potential to interpret considerably more spectra than can be analysed by humans. Machine learning has also been applied to experimental spectroscopies^31–33^. However, previous studies mainly involved the prediction of a scalar value or discrete values in a simple spectrum, such as a chemical shift or peaks in an NMR spectrum^31–33^. Machine learning has not been applied to determine complex spectral features, such as those in ELNES/XANES.

To overcome the limitations of “human-driven” spectral interpretations, we developed a new “data-driven” approach to predict and interpret spectra. Our approach was applied to 46 O-K ELNES/XANES edges of oxide compounds and was successful at predicting and interpreting the spectra. Even non-experts can easily apply our approach to extract information from the ELNES/XANES spectra and utilize this information to obtain new physical insights without theoretical calculations.

## Results and Discussion

### Overview of the data-driven prediction and interpretation method

**Figure 1:**
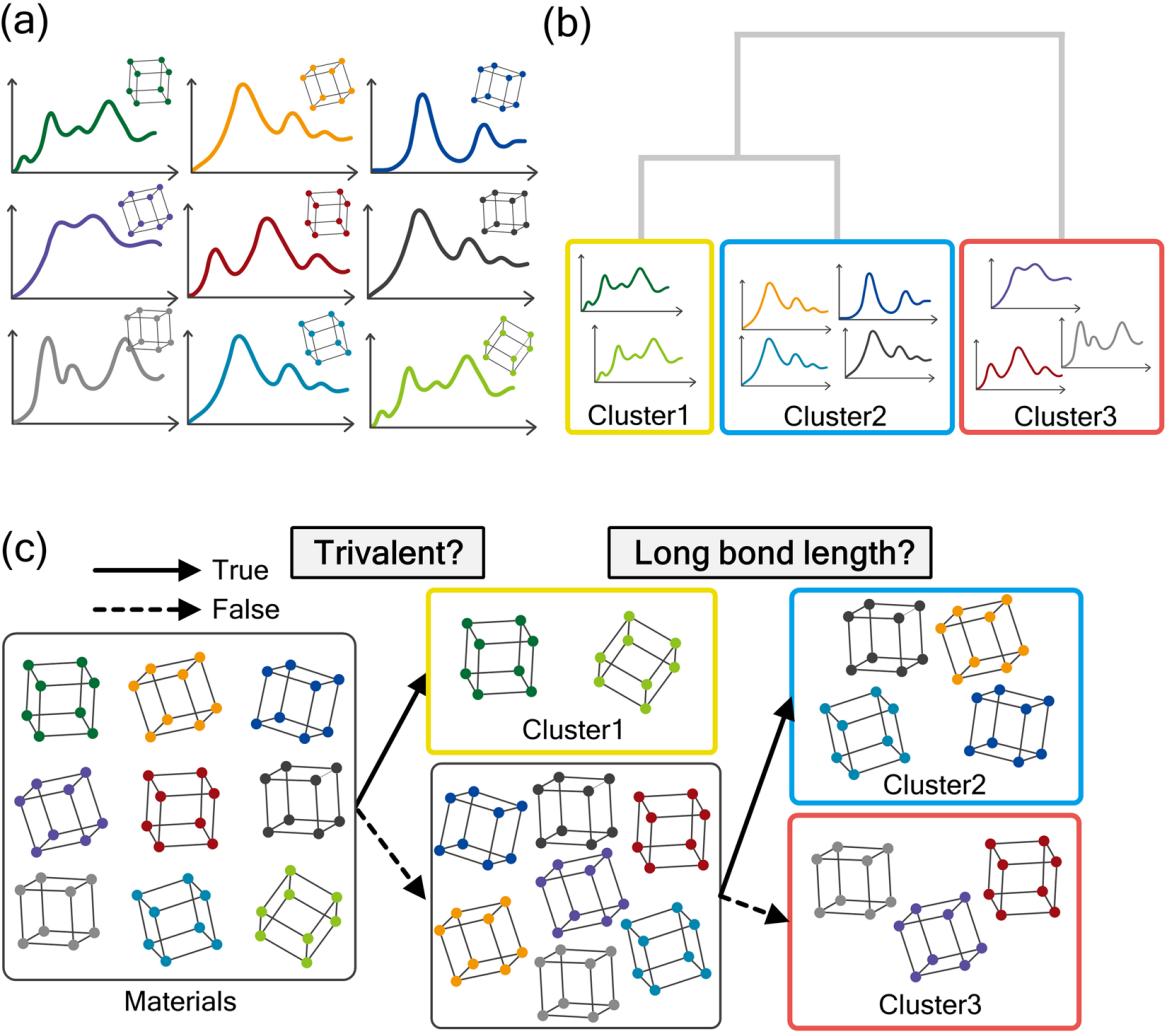
Strategy for the data-driven prediction and interpretation of spectra. Schematics of (**a**) the spectral database and structures, which have one-to-one correspondence, (**b**) spectral clustering, and (**c**) structural classification with the spectral groups as the training data.

The schematic of the proposed “data-driven” spectral analysis is presented in Fig. 1(a–c). Initially, a spectral database, in which each spectrum has a one-to-one correspondence with its atomic structure, is constructed (Fig. 1(a)). As the purpose of this study to establish a “data-driven” approach to predict and interpret spectra, we constructed the spectral database using theoretical calculations. For this purpose, we calculated all spectra with the same parameters, for example, k-points, cutoff energy, and cell size, under the one-particle density functional theory-generalized gradient approximation (DFT-GGA) theoretical framework. After creating the database, the included spectra were divided into groups according to their “similarity” using hierarchical cluster analysis, which divides the spectral data into tree-shaped clusters called “dendrogram” (Fig. 1(b)).

In the final step, the materials are classified into groups with a decision tree based on information such as the bond length, coordination number, group in the periodic table, and valency. As schematically shown in Fig. 1(c), materials are classified using material information such as “trivalent”: true/false and “long bond length”: true/false. The most important aspect of our method is the classification of the materials into the spectral groups, which are used as the training labels for classification of the materials. As the decision tree for materials is based on the spectral groups, this decision tree establishes a correlation between the material information and spectral features. In the decision tree for material information, the branch points provide the “characteristic” descriptors for classifying the spectra, as discussed later.

**Figure 2:**
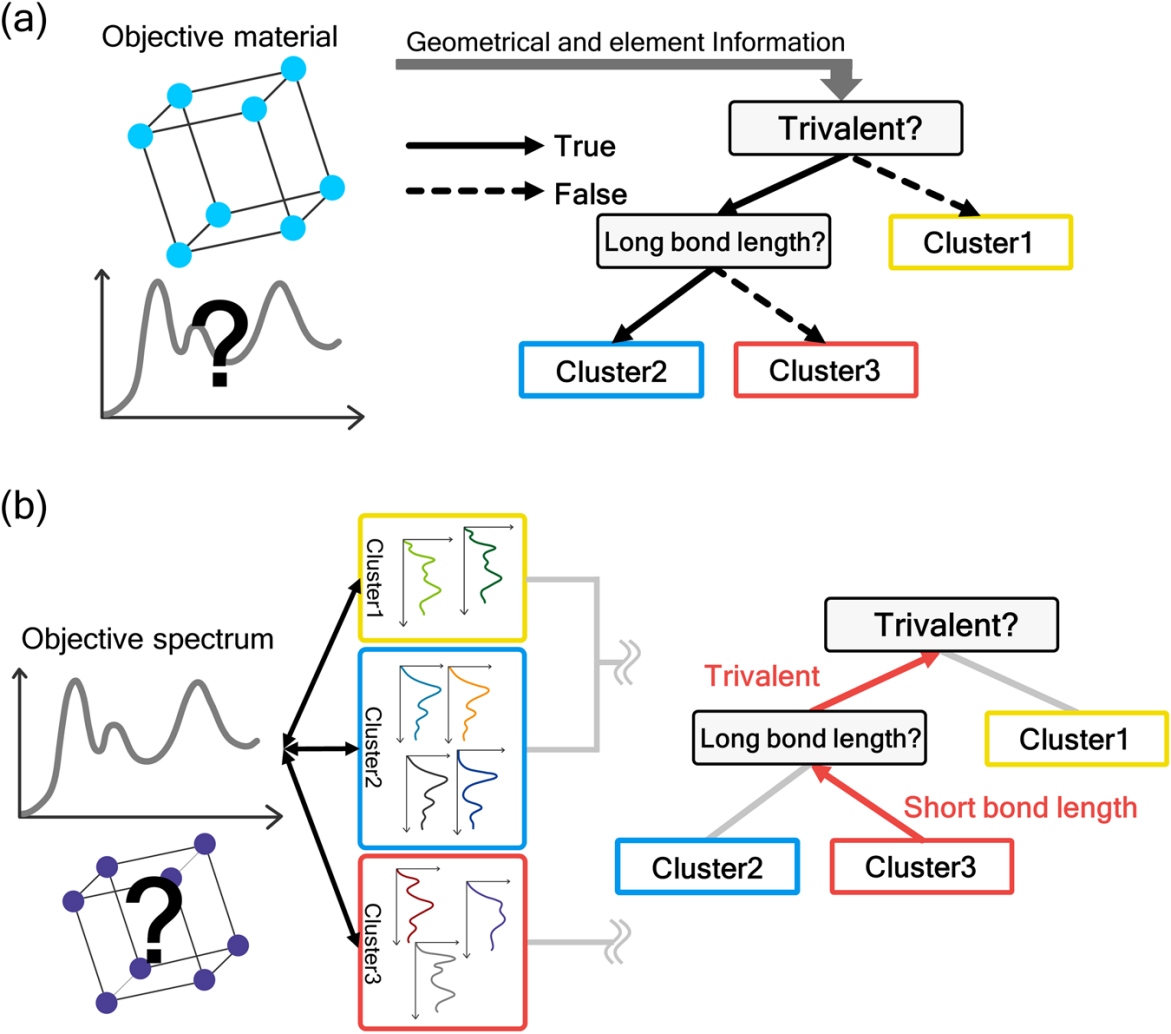
Conceptual schemes of (**a**) “downstream” spectral prediction and (**b**) “upstream” spectral interpretation.

Two trees, related to the spectrum and material information, which are correlated, are constructed in our approach. After the construction of these two trees, the prediction and interpretation of the ELNES/XANES spectra can be performed. The strategy for the prediction and interpretation of the spectra is schematically depicted in Fig. 2.

Figure 2(a) shows the prediction method. For instance, consider a situation in which the atomic configuration of an objective material is known but its ELNES/XANES spectral profiles are unknown. Such situations often occur because we generally speculate (predict) the spectral profile prior to making observations to validate the experiment and sample conditions.

For the prediction, we apply geometric and element information, such as the bond length, angles, and valence state, to the material information decision tree. Hence, the decision tree becomes a true/false diagram (flowchart), for example, the true/false diagrams for trivalence and the bond length, as shown in Fig. 2(a). Moving down the true/false diagram using material (geometric and elemental) information, we arrive at any one of the labels, clusters 1, 2, or 3, as shown in Fig. 2(a); as the decision tree for material information is correlated to that of the spectrum, the objective spectrum should be similar to that in the cluster. This “downstream” method corresponds to the “prediction” of the unknown spectrum of a known structure.

Next, the interpretation method is explained. The “interpretation” of the spectrum is first defined. ELNES/XANES spectra reflect the atomic and electronic structures; thus, the “interpretation of the ELNES/XANES spectrum” corresponds to the determination of the relationship between the spectrum and the atomic and electronic structures.

Consider a situation in which a spectrum of an unknown area/material is observed. In this case, we need to interpret the spectrum, i.e., the relationship between the spectral profile and material information, such as the bond length, coordination number, etc., needs to be determined. For such interpretation, we again use two trees but move up the decision tree.

The strategy for interpretation is schematically shown in Fig. 2(b). For the ELNES/XANES spectrum of an unknown area/material, the cluster in the spectrum tree that is the most similar to the observed spectrum is first determined. In this study, the similarity is estimated by the cosine distance between a pair of spectra, which is often used for measuring the spectral similarity^34,35^. By measuring the cosine distances between the objective spectrum and all the other spectra, the spectrum with the shortest cosine distance is considered to be most similar to the objective spectrum.

In the example in Fig. 2(b), assume that the observed spectrum was the most similar to a spectrum in Cluster 3. Then, moving up the material information tree starting from Cluster 3, geometric and elemental information, such as the bond length, valency, etc., can be obtained from the branching points. Then, the spectrum is interpreted using the atomic and electronic-structure information. In the case of Fig. 2(b), we can establish that the observed spectrum is obtained from a material with a “short bond length” and is “trivalent”.

The branching points in a decision tree play a critical role in the interpretation. When the most similar spectral cluster has been determined, the objective material can be expected to have a structure similar to the materials in the cluster. However, the “characteristic” information of the corresponding cluster cannot be obtained. The branching points in the decision tree provide the “characteristic” information of the materials in the corresponding cluster. We applied this two-trees method to the prediction and interpretation of O-K ELNES/XANES edges. The details of the theoretical calculations and machine learning methods for clustering and classification are described in the Methods section.

In this study, we focus on the oxygen-K (O-K) edges of monometal oxides because these edges can be easily calculated using the one-particle method based on DFT under the GGA framework; the O-K edge provides all information for the conduction band.

In this study, we calculated 46 O-K edges of oxide materials, including 14 monometal oxides (listed in Table 1) and 32 SiO_2_ polymorphous (listed in Table 2). These 14 monometal oxides were selected because they do not have “complex” electronic structures, such as partially occupied 3d-orbitals or magnetism. SiO_2_ was selected because it has several polymorphs.

**Table 1:** List of oxides and their crystal structures.

Metal oxide	Crystal structure
Li_2_O	antifluorite
BeO	rock salt
Na_2_O	antifluorite
MgO	rock salt
Al_2_O_3_	corundum
SiO_2_	β-cristobalite
CaO	rock salt
TiO_2_	rutile, anatase
Ga_2_O_3_	β-form
Y_2_O_3_	bixbite
ZrO_2_	fluorite
In_2_O_3_	corundum
SnO_2_	rutile

TiO_2_ has two types of polymorphs.

**Table 2:** List of SiO_2_ polymorphs, their space groups and names.

Label	Space group	Name
Polymorph 1	F4_1_/d$\bar{3}$2/m	β-Cristobalite
Polymorph 2-1, 2	P6_3_/m2/m2/c	Tridymite
Polymorph 3-1, 2, 3, 4	P6/m2/c2/c	Zeolite
Polymorph 4	P4_2_/n$\bar{3}$2/m	—
Polymorph 5-1, 2	R32	—
Polymorph 6-1, 2	F2/d2/d2/d	—
Polymorph 7-1, 2, 3	C12/c1	—
Polymorph 8-1, 2, 3, 4, 5	C12/c1	Coesite
Polymorph 9	P3_1_21	α-Quartz
Polymorph 10	P3_2_21	—
Polymorph 11	I$\bar{4}$2d	—
Polymorph 12	P4_1_2_1_2	α-Cristobalite
Polymorph 13	P6_2_22	β-Quartz

The second number in the label represents the non-equivalent oxygen sites in the unit cells of the respective polymorphs.

The 14 O-K edge spectra of the monometal oxides were used to demonstrate our method. The actual interpretation and prediction were performed using the 32 O-K edge spectra of SiO_2_ polymorphous.

### Demonstration using the O-K edges of monometal oxides

**Figure 3:**
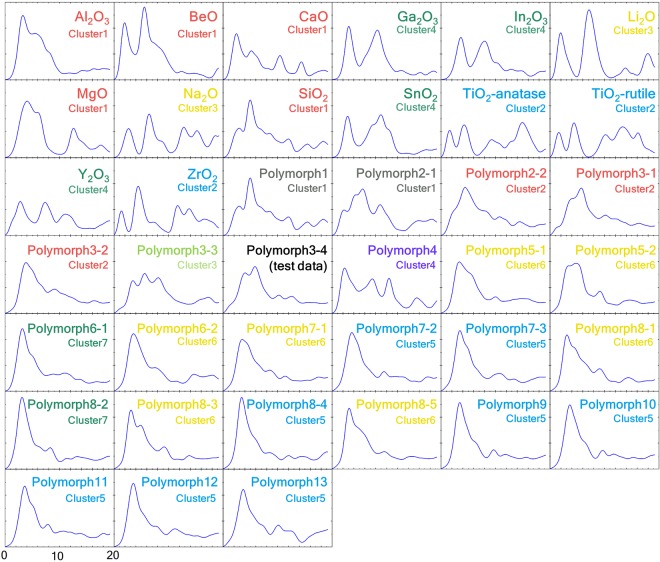
Calculated spectra in this study. O-K edge of 14 oxides and 25 SiO_2_ polymorphs. The label colours correspond to those in the respective dendrograms and decision trees.

**Figure 4:**
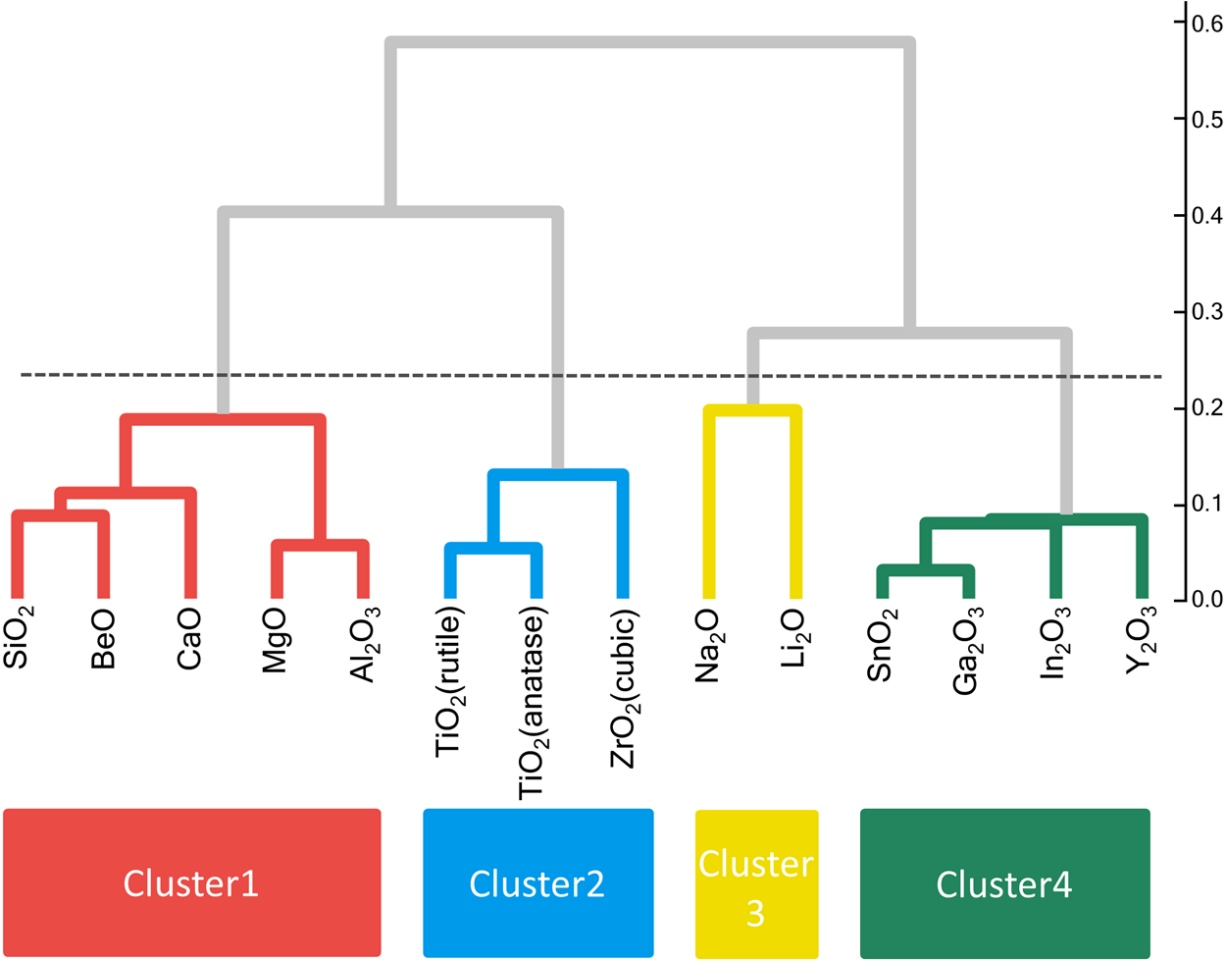
Dendrogram based on the spectral similarities of the 14 metal oxide O-K edges. The axis on the right represents the cosine distances.

Prior to the actual interpretation and prediction, we demonstrate the construction of the two trees. Our approach was first applied to the O-K edges of 14 monometal oxides. All calculated spectra are depicted in Fig. 3. The cluster analysis result for the spectra forms a dendrogram, as shown in Fig. 4. Starting with no links at the bottom of the dendrogram, similar spectra and/or clusters gradually merge, moving upward in the dendrogram. By cutting the dendrogram horizontally at a certain level, a set of clusters can be obtained. The selection of the cutting level is arbitrary; we selected the level indicated by the dashed line in the dendrogram, resulting in four clusters, Cluster 1 to Cluster 4. These four clusters have certain characteristic features, for example, Cluster 3 was composed of alkali metal oxides. We attempted to interpret these features using the decision tree.

**Figure 5:**
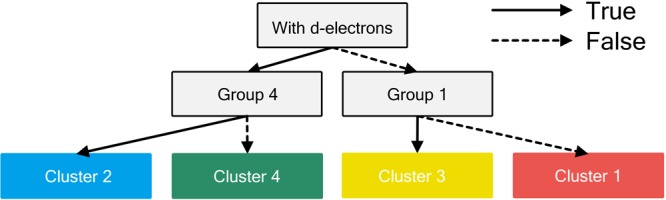
Decision tree based on the training labels of Fig. 4.

Eight parameters were selected as descriptors for the decision tree: 1) valence of the cation, 2) group and 3) period in the periodic table, coordination number of the 4) anion and 5) cation, and the number of 6) s, 7) p, and 8) d electrons of the cation in the valence and semicore states. Figure 5 shows the constructed decision tree based on supervised learning; the decision tree divided the 14 oxides into two subsets based on whether they initially had d electrons and whether the “1^st^ element or not” divided a subset and the “4^th^ element or not” divided the other. The two constructed trees, shown in Figs 4 and 5, were used for interpretation.

For interpreting the spectra, we move up the decision tree, as mentioned above; this provides information on each cluster, for example, Cluster 3 was “with 1^st^ elements” and “without d electrons”, which agree well with what we were previously considered from the dendrogram.

As the demonstration, we used the elemental information of the 14 oxides. However, the relationship between the spectral features and certain geometric information, such as the bond length and coordination number, are often important for the interpretation of ELNES/XANES spectra. To perform the actual interpretation and prediction of the ELNES spectra, we apply this method to the 32 O-K edge spectra of SiO_2_ in the next section.

### Interpretation and prediction of the O-K edges of SiO_2_ polymorphous

We considered 13 polymorphs and 7 virtual polymorphs of SiO_2_, the space groups of which are listed in Table 2. As some polymorphs have several oxygen sites, namely, 2-1, 2; 3-1~4; 5-1, 2; 6-1, 2; 7-1~3; 8-1~5, all were calculated separately. Thus, a total of 32 O-K edge spectra were obtained. The O-K edge of SiO_2_ was selected because although the composition is fixed, the atomic configuration is different and the spectral features can be interpreted using geometric information, such as Si-O bond length and Si-O-Si bond angle. Among them, excluding 7 virtual structures (described later) and randomly selected test data, polymorph 3-4, 24 spectra were used for hierarchical clustering and creating the decision tree.

**Figure 6:**
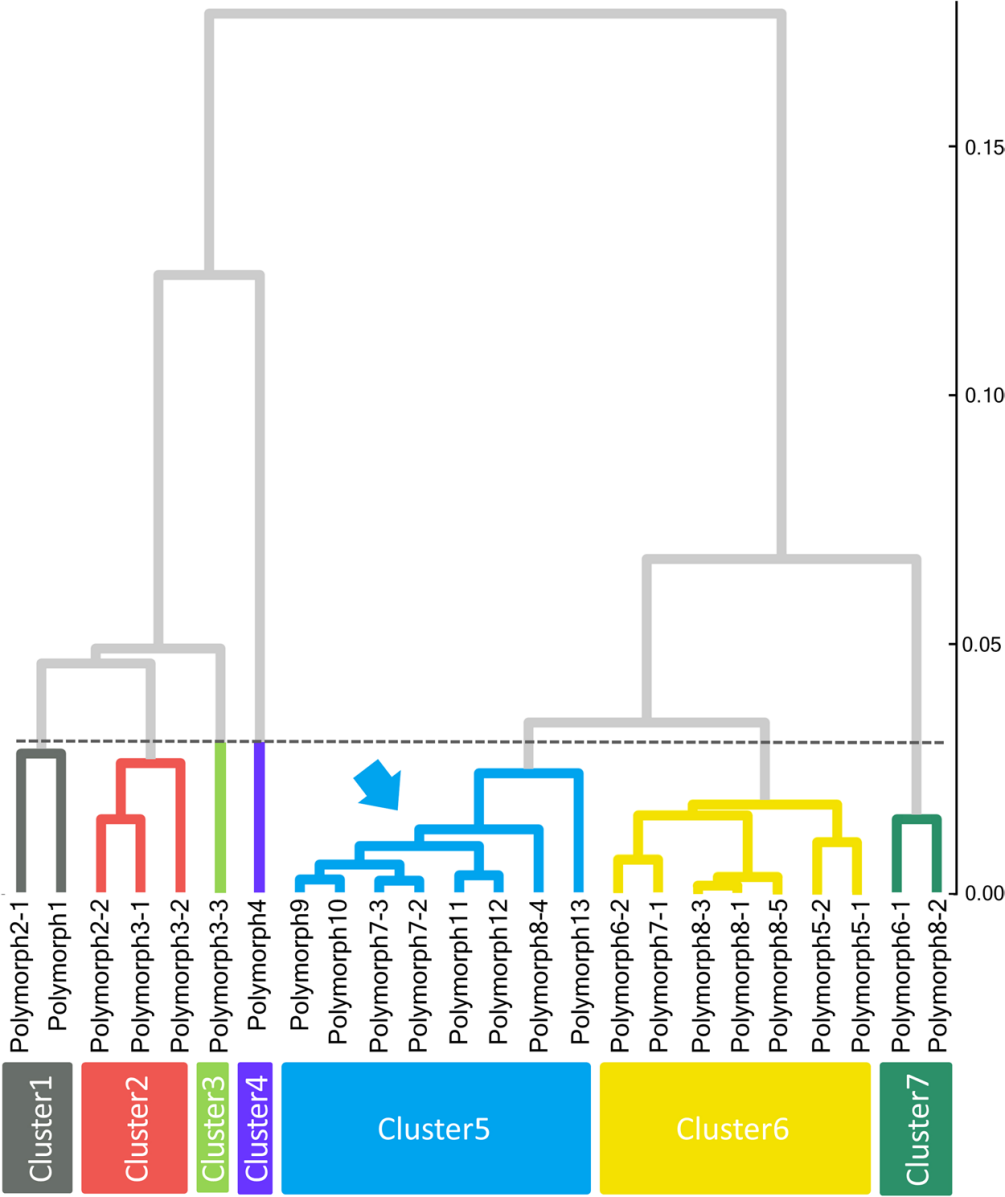
Dendrogram based on the spectral similarities of the 24 O-K edges of the SiO_2_ polymorphs. The axis on the right represents the cosine distances.

The spectral features of the polymorph O-K edges are shown in Fig. 3, which clearly exhibit a variety of spectral profiles. The cluster dendrogram for these 24 spectra is depicted in Fig. 6. At the threshold level in Fig. 6, which was not set arbitrarily but in data-driven manner as described below, seven clusters were generated. Cluster 4, that is, polymorph 4, is separated from the other spectra at a higher level, indicating that its spectrum is highly dissimilar to those of the other polymorphs. In fact, the spectral features of polymorph 4 differ considerably from those of the others (Fig. 3). This result is supported by the fact that the crystal structure of polymorph 4 has higher symmetry than the other polymorphs. Polymorphs 7-2, 7-3, 8-4, 9, 10, 11, 12, and 13 formed the large blue group, as their spectral features are very similar to each other (Fig. 3).

We next focus on the blue group, namely, Cluster 5 (indicated by the blue arrow in Fig. 6). Spectra in this group commonly include a large peak at the spectrum threshold, followed by small peaks, as shown in Fig. 3 (spectra with blue labels). Their features are visually very similar to those of the spectra in the yellow and green groups, namely, Cluster 6 and 7. However, our approach could tell that these three groups have some imperceptible differences. A detailed comparison revealed a small distinction, wherein the peaks of the blue group were slightly sharper than those of the other two groups. The sharper profile of Cluster 5 is ascribed to the more symmetric structures of the included materials. Most of the materials in Cluster 5 have a single oxygen site, whereas those in Cluster 6 and 7 have multiple oxygen sites in their unit cells. Moreover, the spectra in Cluster 7 also have a sharp first peak, as in the spectra in Cluster 5, but the position of the first peak is slightly different from that in Cluster 5. These small spectral differences may be difficult to be found by the human eye, but the present classification method has the potential to discern such small differences.

These spectral features are very difficult to detect by one-to-one comparison, and their categorization by the human eye is difficult. However, the proposed data-driven method can determine their differences and categorize numerous spectra.

**Figure 7:**
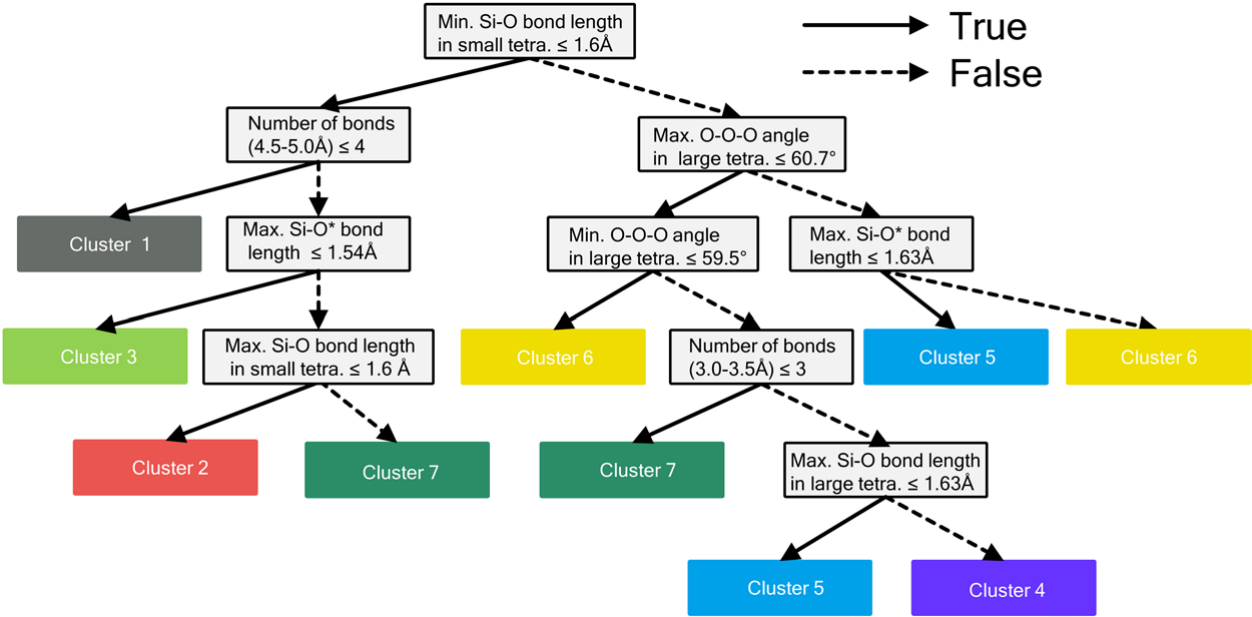
Decision tree based on the training labels of Fig. 6. Each grey rectangle at the branching point represents a division rule. The solid and dashed lines indicate “true” and “false,” respectively.

Next, we constructed the decision tree based on the structural information of these polymorphs. For this, we selected 32 geometric features as descriptors for creating the decision tree. These 32 descriptors are listed in Table 3. As previously mentioned, the construction of the decision tree was performed by supervised learning. Figure 7 depicts the constructed decision tree based on the threshold level indicated by the dashed line in Fig. 6. To determine the threshold line, we evaluated the accuracy rate for the training data at each level. As a result, decision trees with higher thresholds than the current line showed 100% correct prediction for the training data set.

**Table 3:** List of descriptors for the SiO_2_ polymorphs.

Thirty-two descriptors for SiO_2_ polymorphous
Number of bonds (1.0–1.5, 2.0–2.5, 2.5–3.0, 3.0–3.5, 3.5–4.0, 4.0–4.5, 4.5–5.0, 5.0–5.5, 5.5–6.0 Å)
Shorter Si-O* bond length
Average Si-O* bond length
Si-O*-Si angle
Longer Si-O bond length
Shorter Si-O bond length
Average Si-O bond length
Volume of larger/smaller tetrahedron
Average O-O-O angle in larger/smaller tetrahedron
Average O-Si-O angle in larger/smaller tetrahedron

O^*^ indicates that the oxygen has a core hole.

To evaluate the constructed decision tree, the test data, namely, polymorph 3-4, was used for prediction and interpretation. First, we attempted to predict the spectrum of the test data (polymorph 3-4). In this case, the geometric information of polymorph 3-4 is known, as summarized in Table 4. As mentioned above, we move down the decision tree (Fig. 7) for prediction, using the geometric information of the polymorph 3-4 site; the decision tree starts from “minimal Si-O bond length in small tetrahedron ≤1.6 Å”. The site for polymorph 3-4 has a minimal Si-O bond length in the small tetrahedron of 1.53 Å; thus, it is “true”. The next true/false decision is the “number of bonds between 4.4–5.0 Å ≤ 4”. For polymorph 3-4, this value is 15; thus, it is “false”. Furthermore, as per the polymorph 3-4 information, listed in Table 4, Cluster 2 (red group) is reached.

**Table 4:** List of the geometric characteristics of polymorph 3-4.

Descriptor	Polymorph 3-4
Minimal Si-O bond length in small tetra.	1.53 Å
Number of bonds (4.5–5.0 Å)	15
Maximum Si-O* bond length	1.60 Å
Maximum Si-O bond length in small tetra.	1.59 Å

**Figure 8:**
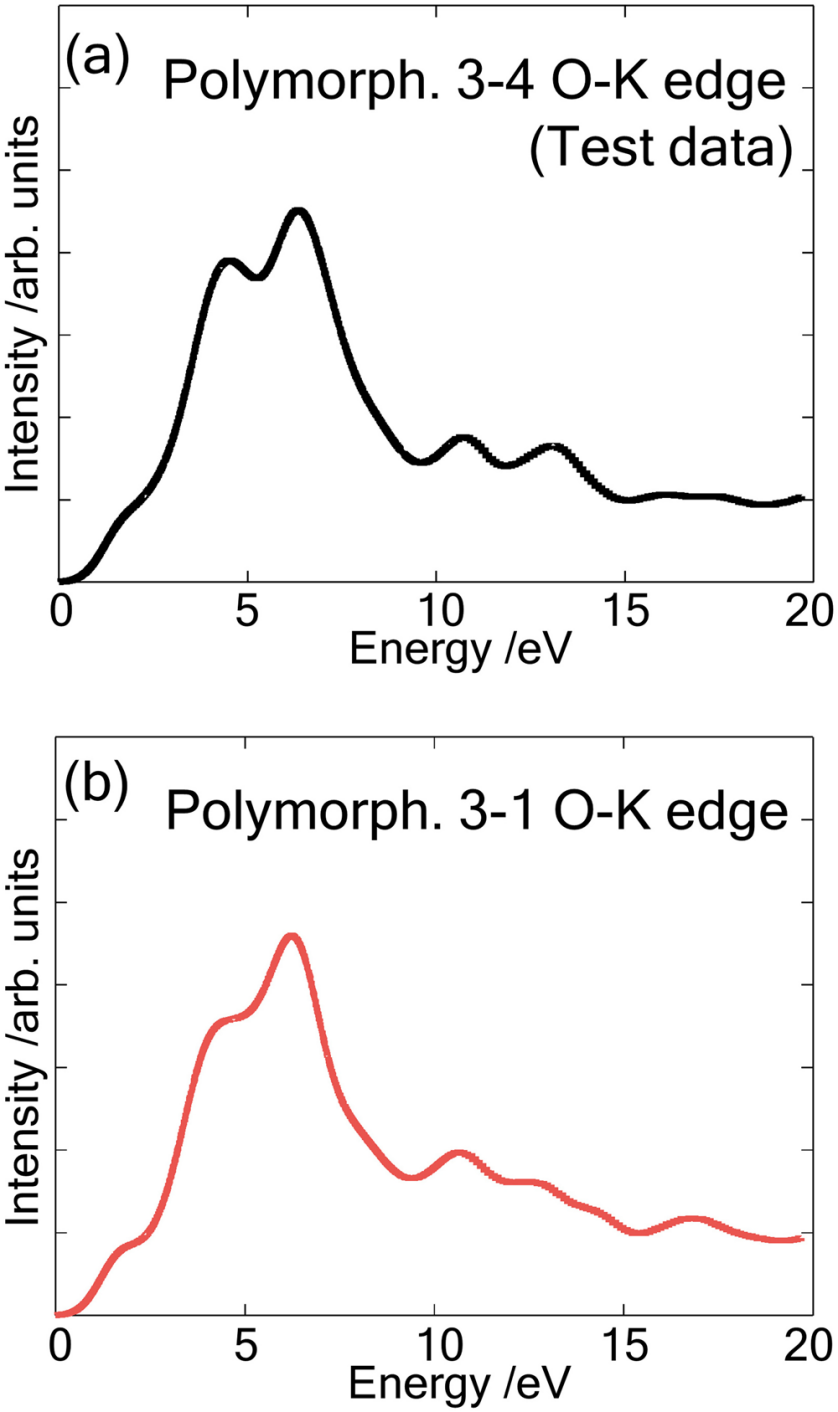
Calculated O-K edge spectra of (**a**) polymorph 3-4 and (**b**) polymorph 3-1.

Based on the geometric information of the site, the proposed method suggests that the spectral features of polymorph 3-4 are similar to those of Cluster 2, which is composed of polymorph 2-2, 3-1 and 3-2. The actual spectrum for polymorph 3-4 is shown in Fig. 8(a) together with the spectrum of polymorph 3-1 (Fig. 8(b)). Our method predicted that the test data belonged to Cluster 2, and indeed the spectral features of polymorph 3-4 is very similar to that for polymorph 3-1, indicating that this method can predict the spectral features correctly.

Next, our method is used for interpreting the spectrum. We consider a different situation in which we know the spectral features of polymorph 3-4 but not the material information. First, we measure the cosine distance between the objective spectrum (in this case, the spectrum of polymorph 3-4) and all other spectra in the database. In this case, the cosine distance to polymorph 3-1 is the least, indicating that this spectrum is most similar to that of polymorph 3-4. Through this process, the class of the objective spectrum can be determined. In this test case, the objective spectrum is categorized as Cluster 2 because polymorph 3-1 belongs to Cluster 2.

The spectrum is interpreted using the decision tree in Fig. 7. Since the objective spectrum is most similar to that of polymorph 3-1, the material information of the objective material is expected to be similar to that of polymorph 3-1 and the other polymorphs in Cluster 2. However, as mentioned above, the “characteristic” features of the materials in Cluster 2 cannot be determined. To obtain the “characteristic” feature, we need to travel up the decision tree in Fig. 7.

We can obtain the initial information, “maximum Si-O bond length in the small tetrahedron ≤1.6 Å”, and the next branch points, “max. Si-O* bond length ≥ 1.54 Å” and “number of bonds between 4.5 to 5.0 Å ≥ 4”, from the decision tree (Fig. 7). This information agrees with the geometric information of polymorph 3-4 (as summarized in Table 4). Thus, we can obtain the “characteristic” features of the materials in Cluster 2 from the branching points of the decision tree, and the spectrum can be interpreted using these “characteristic” features.

**Figure 9:**
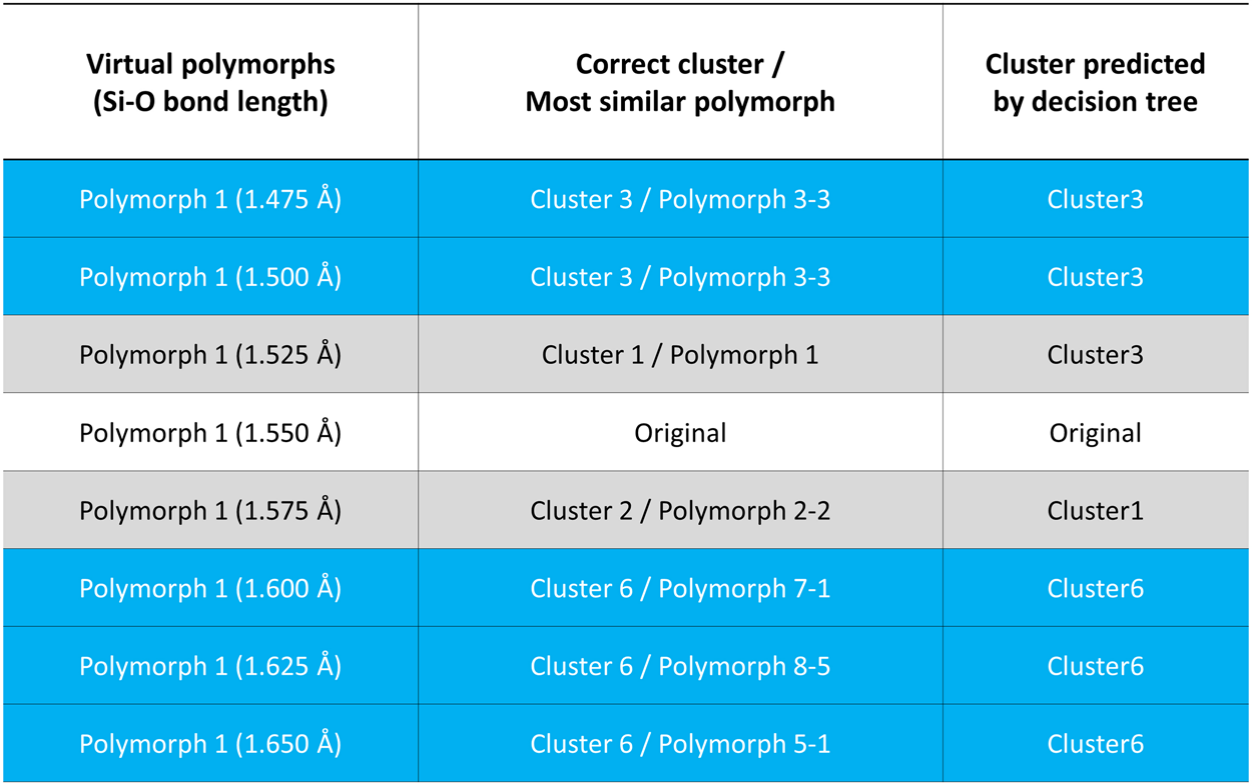
List of virtual polymorphs, correct labels/most similar polymorph, and predicted cluster by the decision tree in Fig. 7. The non-shaded row indicates the original polymorph 1 (β-cristobalite). Blue- and grey-shaded rows indicate successful and failed predictions, respectively.

**Figure 10:**
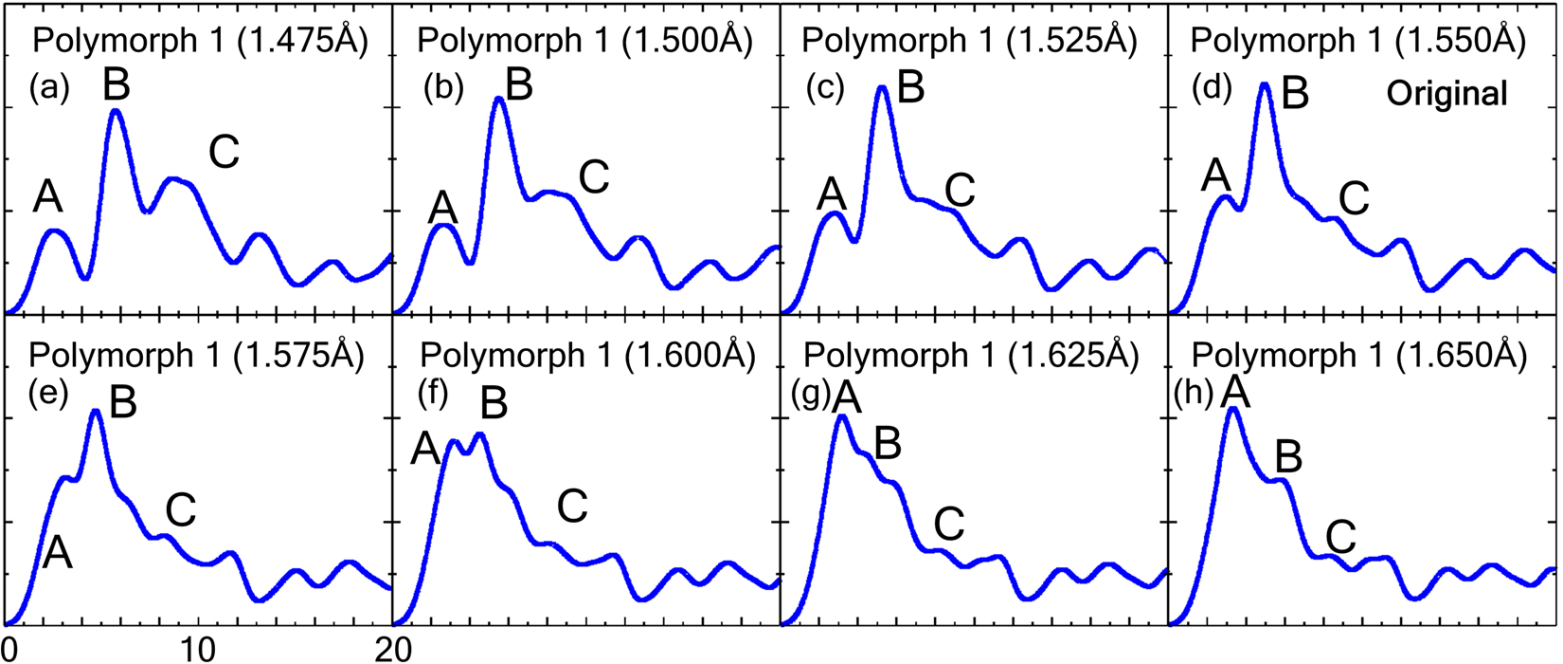
Calculated O-K edge spectra of the original polymorph 1 (d) and seven virtual structures (**a**–**c**) and (**e**–**h**) with the same crystal structure but different Si-O bond lengths.

Finally, to determine the further applicability and limitations of the present method, we applied this method to other SiO_2_ systems. Here, seven virtual structures with the same crystal structure as polymorph 1 (β-cristobalite) but different volumes were constructed, as listed in Fig. 9. Polymorph 1 was selected because of its highly symmetric structure, which allows us to easily investigate the local coordination. The Si-O bond lengths in these models (1.475~1.650 Å) are different from those in the original structure (1.550 Å). Their calculated spectra are shown in Fig. 10. The original spectrum is composed of a sharp peak B with small peaks A and a plateau peak C in the lower and higher energy regions (Fig. 10(d)), where the intensity of peak A gradually decreases/increases with decreasing/increasing Si-O bond length.

The spectra are categorized by their spectral features (middle column in Fig. 9) into Cluster 3, 1, 2, or 6. Based on the Si-O bond length and coordination environment, the cluster category can be predicted using the decision tree in Fig. 7, as demonstrated above in the “prediction” section. The results of the predicted cluster are summarized in the right column in Fig. 9. The present method correctly predicted five of the seven structures (shaded by blue in Fig. 9), whereas the prediction failed for the remaining two structures (shaded by grey in Fig. 9). From the succeeded/failed structures, we found that the present method can correctly predict when the Si-O bond length is largely different from the original, whereas the method failed when the virtual structures are very similar to the original structure.

The success or failure of the prediction can be ascribed to the training data used to construct the decision tree. The decision tree in Fig. 7 was constructed from polymorphs, which commonly have sufficiently different structures, and the tree did not “learn” tiny structural changes, such as a 0.025 Å bond length difference. Thus, structures with small structural changes cannot be predicted correctly using the present decision tree. However, the decision tree works well when the magnitude of the structural change is compatible to that in the polymorph tree.

This result indicates the applicability as well as the limitations of the present method, namely, the prediction/interpretation depends on the training data used to make the two trees. The construction of two trees trained by many polymorphs is enough to predict/interpret relatively large spectral/structural changes. However, training data from virtual structures, as discussed above, should be necessary to predict/interpret very small spectral/structural changes. This indicates that the combined database of both experimental and calculated spectra is important to achieve versatile prediction and interpretation.

## Conclusion

In this study, we proposed a “data-driven” approach for predicting and interpreting ELNES/XANES spectra. Our method is based on the hierarchical clustering and decision tree. The calculated oxygen-K edges of 14 metal oxides and 32 SiO_2_ polymorphs, including 7 virtual structures, were used to demonstrate the proposed method.

With this method, the ELNES/XANES spectra can be interpreted in accordance with material information, such as chemical, elemental, and geometric information. Furthermore, our method was effectively used for predicting the spectral features from the material information.

To establish the proposed methodology, we constructed a spectral database using theoretical calculations. We emphasize that the proposed machine learning method is not spectroscopy-dependent and thus should work well for interpreting and predicting any spectral data, even diffraction, emission, and experimental data. A substantial database of over 300,000 calculated spectra was recently constructed^36^, and therefore, combining this database or others like it with our method allows for versatile and accurate predictions and interpretations. We believe that our method can pave the way for “data-driven” spectral interpretation and prediction.

## Methods

### Construction of the spectral database

The CASTEP^37,38^ code was used for ELNES/XANES calculations, which is based on the first-principles plane-wave basis pseudopotential method. GGA-PBE^39^ was selected as the approximation of the exchange-correlation functional, and the cutoff energy was set to 500 eV. To introduce core-hole effects, an excited pseudopotential was generated and applied to the excited oxygen atom in the supercell. To minimize interactions among excited atoms under periodic boundary conditions, sufficiently large supercells, larger than 10 Å, were used in all cases. The theoretical transition energy was also simulated, similar to a previous study^38^. The oxygen atom with a core hole is denoted “O*”. The O-K edges calculated in this study are shown in Fig. 3.

### Spectral data clustering

Hierarchical clustering^40^ was applied to categorize the spectral data. Initially, each spectrum was assigned to its own cluster. Then, the two most similar clusters were combined into one cluster. The clustering process was repeated until the number of clusters was unity. The spectral similarity was estimated using the cosine distance, and the clustering linkage schemes “completed” the method. In this study, as we directly compare the spectral feature itself, the onsets of all spectra were aligned. To estimate the position of the onset, the double differential of the spectrum was used.

### Decision tree for material information

A decision tree visualizes the classification or regression results by a tree structure. By repeatedly dividing the data into two or more subsets, the decision tree is composed of certain subsets with labels, which are the same as the labels in the training data. As this is a type of supervised learning method, training labels are necessary. As described above, the labels obtained in the spectral clustering were used as the training data. The classification and regression tree (CART) algorithm^41,42^ was used for training.
